# Influenzalike Illness Among Homeless Persons

**DOI:** 10.3201/eid1207.060217

**Published:** 2006-07

**Authors:** Scott J. Bucher, Philip W. Brickner, Richard L. Vincent

**Affiliations:** * St. Vincent's Hospital-Manhattan, New York, New York, USA

**Keywords:** Homeless persons, influenza, human, vaccination, letter

**To the Editor:** We report rates of influenzalike illness (ILI) and influenza vaccination among homeless persons at 3 shelter clinics in New York City examined from 1997 through 2004. Little is understood regarding the prevalence and transmission of influenza among the homeless (*1*). Further inquiry on this topic is timely because of concern over a possible influenza pandemic, because of US goals to increase vaccination rates among high-risk groups (*2*), and because of the potential threat to persons who live and work in shelters. Homeless shelters are paradigmatic congregate settings and thus likely sites for transmission of airborne pathogens such as influenza viruses and tubercle bacilli, shown in part by numerous tuberculosis outbreaks among the homeless (*3*).

Homeless persons experience high rates of pneumonia (*4*) and related death (*5**,**6*). This outcome indicates that the homeless also have high rates of influenza because pneumonia is a common complication of influenza. Depending upon patient's age and sex, death rates attributed to pneumonia or influenza among homeless adults ranged from 1.6 to 6.3 (95% confidence interval 0.4–24.1) in one study (*7*). The New York City Departments of Health and Mental Hygiene and Homeless Services reported in December 2005 that 1% of hospitalizations and 3.4% of deaths of homeless adults in New York City from 2001 to 2003 were caused by influenza or pneumonia (*8*).

We analyzed 4,319 medical charts of persons who received medical services in 3 New York City homeless shelter clinics during influenza seasons (i.e., October 1 through May 30) from 1997 through 2004. This study was approved by the St. Vincent's Hospital Research Committee and Institutional Review Board. This analysis identified 59 recorded cases of ILI, defined as temperature >100°F (37.8°C) and cough, sore throat, or both (Table). ILI is accepted as an indicator of influenza by the Centers for Disease Control and Prevention and others (*9*).

**Table:** Cases of influenzalike illness (ILI) among homeless persons by influenza season, New York City, 1997–2004

Season	Shelter 1			Shelter 2			Shelter 3			Total		
	No. cases	No. patients seen	% patients seen with ILI	No. cases	No. patients seen	% patients seen with ILI	No. cases	No. patients seen	% patients seen with ILI	No. cases	No. patients seen	% patients seen with ILI
1997–98	5	284	1.8	3	221	1.4	3	202	1.5	11	707	1.6
1998–99	4	363	1.1	5	197	2.5	5	240	2.1	14	800	1.8
1999–00	2	170	1.2	1	186	0.5	4	248	1.6	7	604	1.2
2000–01	1	198	0.5	2	206	1.0	4	227	1.8	7	631	1.1
2001–02	2	202	1.0	2	122	1.6	1	258	0.4	5	582	0.9
2002–03	2	196	1.0	1	136	0.7	1	218	0.5	4	550	0.7
2003–04	6	152	3.9	1	157	0.6	4	235	1.7	11	544	2.0
Total	22	1,565	1.4	15	1,225	1.2	22	1,628	1.4	59	4,418	1.3

The overall medical chart review also showed that less than one fourth of all persons examined and one third of those >65 years of age had evidence of influenza vaccination noted in their charts. Vaccinations are available from many sources, but those given at shelter clinics accounted for a large percentage, and vaccination rates varied widely by homeless shelter clinic site.

This study has some limitations. Because vaccinations are offered at numerous health centers, rates of vaccination based on the medical charts we studied may be underestimated. Moreover, since only those homeless persons at shelters who attended the medical clinic provided data, the findings cannot be used to make generalizations regarding ILI or influenza vaccination rates among the general population of the shelters. Nonetheless, these numbers can serve as a basis for more rigorous inquiry.

The implementation of an appropriate public health response is critical in maintaining the health of homeless persons. Controlling influenza transmission within shelters may benefit the broader public in the same way that reducing the rates of tuberculosis among homeless persons is regarded as essential in preventing transmission to the general population.

The decision to receive an influenza vaccination is influenced by many factors. These factors include concern with related side effects, belief that the vaccine is not required, previous bad reactions, dislike of injections, and doubts about vaccine efficacy (*10*). Understanding how these factors affect vaccination rates among the homeless would be valuable in planning healthcare interactions and quality improvements. Similarly, since the New York City Departments of Health and Mental Hygiene and Homeless Services recommend that influenza immunizations be provided to all sheltered homeless adults and shelter staff (*8*), further inquiry would help determine the risk-benefit balance of such an approach.
